# Carbon/Nitrogen Imbalance Associated with Drought-Induced Leaf Senescence in *Sorghum bicolor*

**DOI:** 10.1371/journal.pone.0137026

**Published:** 2015-08-28

**Authors:** Daoqian Chen, Shiwen Wang, Binglin Xiong, Beibei Cao, Xiping Deng

**Affiliations:** 1 College of Life Sciences, Northwest A&F University, Yangling, Shaanxi, 712100, China; 2 State Key Laboratory of Soil Erosion and Dryland Farming on the Loess Plateau, Institute of Soil and Water Conservation, Northwest A&F University, Yangling, Shaanxi, 712100, China; 3 Institute of Soil and Water Conservation, Chinese Academy of Sciences and Ministry of Water Resources, Yangling, Shaanxi, 712100, China; 4 College of Natural Resources and Environment, Northwest A&F University, Yangling, Shaanxi, 712100, China; Universidade Federal de Viçosa, BRAZIL

## Abstract

Drought stress triggers mature leaf senescence, which supports plant survival and remobilization of nutrients; yet leaf senescence also critically decreases post-drought crop yield. Drought generally results in carbon/nitrogen imbalance, which is reflected in the increased carbon:nitrogen (C:N) ratio in mature leaves, and which has been shown to be involved in inducing leaf senescence under normal growth conditions. Yet the involvement of the carbon/nitrogen balance in regulation of drought-induced leaf senescence is unclear. To investigate the role of carbon/nitrogen balance in drought-induced senescence, sorghum seedlings were subjected to a gradual soil drought treatment. Leaf senescence symptoms and the C:N ratio, which was indicated by the ratio of non-structural carbohydrate to total N content, were monitored during drought progression. In this study, leaf senescence developed about 12 days after the start of drought treatment, as indicated by various senescence symptoms including decreasing photosynthesis, photosystem II photochemistry efficiency (Fv/Fm) and chlorophyll content, and by the differential expression of senescence marker genes. The C:N ratio was significantly enhanced 10 to 12 days into drought treatment. Leaf senescence occurred in the older (lower) leaves, which had higher C:N ratios, but not in the younger (upper) leaves, which had lower C:N ratios. In addition, a detached leaf assay was conducted to investigate the effect of carbon/nitrogen availability on drought-induced senescence. Exogenous application of excess sugar combined with limited nitrogen promoted drought-induced leaf senescence. Thus our results suggest that the carbon/nitrogen balance may be involved in the regulation of drought-induced leaf senescence.

## Introduction

Drought stress triggers various plant responses, which affect numerous plant systems ranging from gene expression patterns to physiological metabolism to growth and development. One effect of drought stress is leaf senescence, which helps to reduce water loss at the whole-plant level and to remobilize nutrients from the senescing leaves to younger leaves or sink organs; this plays an important role in plant survival. However, drought-induced senescence also leads to reductions in canopy size and photosynthesis rate as well as critical decreases in crop yield after the drought has been eliminated [1,2]. Currently, most research on the signals involved in the onset of drought-induced leaf senescence has focused on the role of phytohormones, especially cytokinin (CK) and abscisic acid (ABA) [3,4], along with reactive oxygen species (ROS) [5].

The availability of carbon (C), especially in its carbohydrate form, and nitrogen (N) are important factors in the regulation of plant metabolism and development [6]. In addition to the individual importance of C and N, the ratio of C metabolites to N metabolites in the cell, which is referred to as the C/N balance, is also important for regulation of plant growth and development [6,7,8]. Analysis of growth at different C:N ratios in *Arabidopsis* has revealed that the C/N balance, rather than C or N alone, plays a predominant role in seedling growth regulation, storage lipid remobilization and photosynthetic gene expression [8]. Global gene expression analysis of *Arabidopsis* responses to a matrix of C:N treatments confirms the proposed importance of combined carbon and nitrogen (CN)-signaling in plants [9]. In addition to its role in early seedling growth, C/N balance also plays an important role in regulating leaf senescence under normal conditions. Leaf senescence can be triggered by high C and low N availability (a typical C/N imbalance)[7,10].

Prolonged drought treatment generally results in sugar accumulation and a decrease in leaf N content, leading to C/N imbalance, which is reflected in the increased C:N ratio in mature leaves. Soluble sugars, especially glucose and fructose, accumulate together with other osmolytes during drought. This phenomenon provides protection against structural and functional damage caused by dehydration and is considered to be a form of plant osmotic adjustment to water deficit [11,12]. Nutrient deficiencies (especially N deficiency) are an intrinsic feature of water deficits in natural and controlled environments [13,14]. Under drought conditions, the loss of transpiration and turgor causes a decrease in NO_3_^-^ absorption by the roots and a reduction in transport from the roots to the leaves [15,16]. Drought stress also restricts the ability of plants to reduce and assimilate N through the inhibition of enzymes involved in N metabolism, such as nitrate reductase and glutamine synthetase [13,17].

Drought stress generally results in a C/N imbalance, and a C/N imbalance typically occurs in association with leaf senescence. To date, however, it was not clear whether C/N balance was involved in the regulation of drought-induced leaf senescence. This research investigated the possible role of C/N balance in regulating drought-induced leaf senescence. Sorghum (*Sorghum bicolor*) seedlings were subjected to a progressive soil drought treatment. The symptoms of leaf senescence (including photosynthetic rate, maximum efficiency of photosystem II photochemistry (Fv/Fm), chlorophyll loss and senescence marker gene expression) and the C:N ratio (indicated by the ratio of non-structural carbohydrate to total N content) were monitored during drought stress. The influence of the exogenous C:N ratio on leaf senescence in detached leaves was also assayed.

## Materials and Methods

### Plant Growth Conditions and Drought Treatments

**Fig 1:**
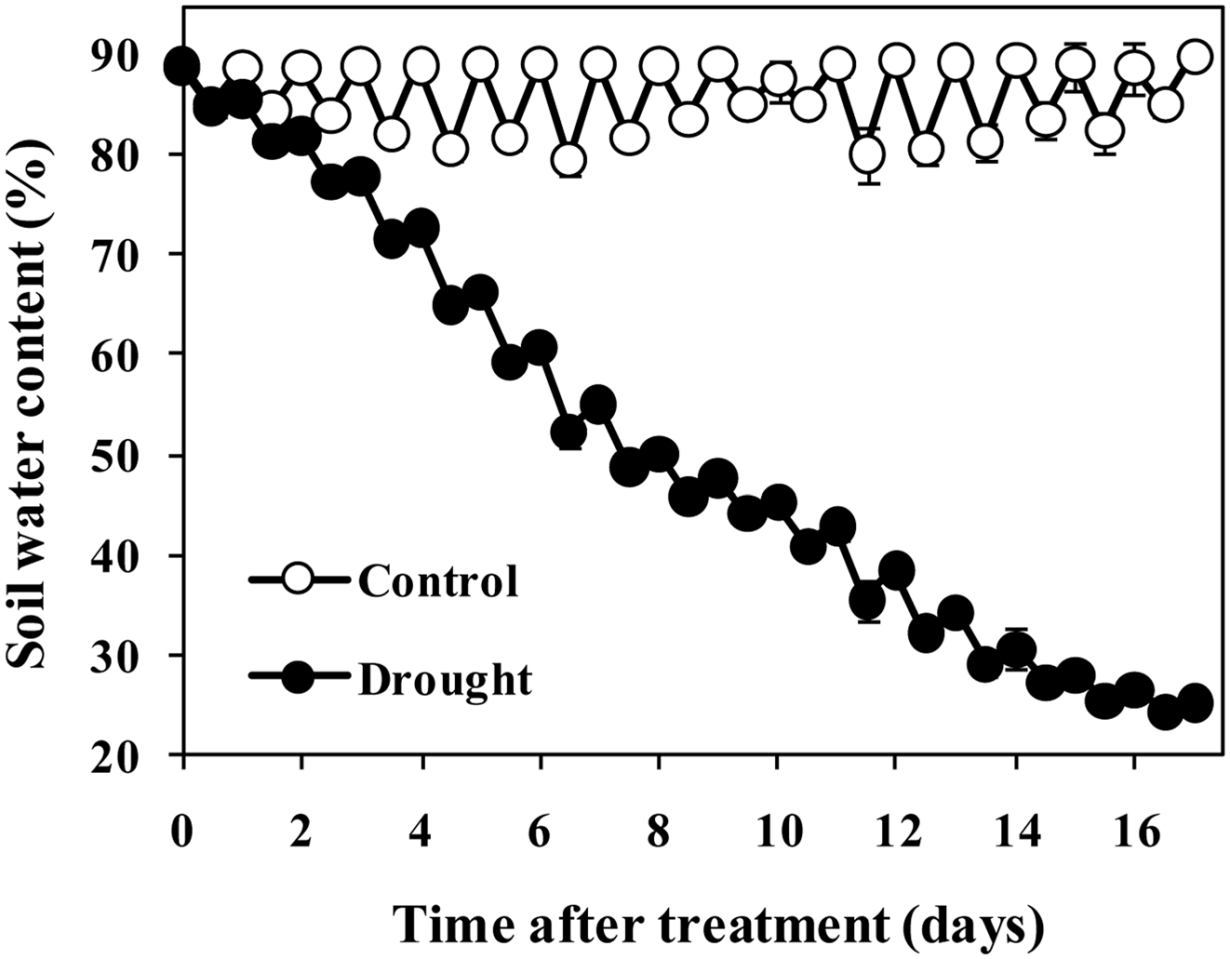
Changes in soil water content during drought progression. Data represent the mean ± SD (n = 30).

Seeds of sorghum [*Sorghum bicolor* (L.) Moench. cv. Gadambalia] were sterilized with 1% sodium hypochlorite for 10 min and then washed with distilled water four times. After sterilization, six seeds were sown per plastic pot filled with 15 kg loessial soil collected from the upper 20 cm of a private cultivated field. Soil collection was permitted by the owner of the land and we confirmed that it did not affect endangered or protected species. The experiment was conducted in a growth chamber with a 14 h photoperiod and day/night temperatures of 28/23°C. Photosynthetic photon flux density was 600 μmol m^−2^ s^−1^ and relative humidity was 40–50%. The seedlings were thinned to two seedlings per pot when the third leaves were fully expanded. When the eighth leaves were fully expanded, a natural progressive drought was imposed by withholding watering based on daily measurements of pot weight. Soil water content was calculated according to pot weight and expressed as a percent maximum pot capacity [18]. For the control treatment, soil water content was maintained between 80% and 90% throughout the experiment; for the drought treatment, the water content of the soil was allowed to decrease progressively. Soil water content under drought conditions dropped to 66.3%, 50.3%, 38.6%, 30.8%, and 26.7%, respectively, on days 5, 8, 12, 14 and 16 (Fig 1). The eighth leaves were sampled during the drought progression and all of the fully expanded leaves above the eighth leaf were sampled at the end of treatment.

### Leaf Water Potential

The water potential of the eighth leaf was measured using a pressure chamber (Model 3500, Soilmoisture Corp., Santa Barbara, CA, USA) in the early morning before lamps were turned on, based on the protocol of Liu et al. [19]. Each treatment includes five replications.

### Leaf Relative Water Content

The eighth leaves were removed and weighed immediately to obtain their fresh weight (FW). Turgid weight (TW) was determined after leaf segments had been immersed in distilled water for 6 h, and dry weight (DW) was measured after leaf segments had been dried at 70°C in an oven for 24 h. The relative water content (RWC) was calculated as follows: RWC = [(FW-DW) / (TW-DW)] ×100. Each treatment includes five replications.

### Photosynthetic Rate

The photosynthetic rates were measured between 9:00 h and 11:00 h using a portable photosynthesis system (Li-6400; LI-COR Inc., Lincoln, NE, USA). The eighth leaf was placed in the chamber at a photon flux density of 1000 μmol m^-2^ s^-1^; the flow rate through the chamber was 500 μmol s^-1^ and leaf temperature was 28°C. The ambient CO_2_ concentration was approximately 410 μmol CO_2_ mol^-1^ air, and vapor pressure deficit was maintained at approximately 2.0 kPa. Each treatment includes five replications.

### Chlorophyll Fluorescence

Fv/Fm in individual leaves was analyzed using a pulse amplitude modulated chlorophyll fluorescence system (Imaging PAM, Walz, Effeltrich, Germany) according to the method of Xu et al. [20]. The eighth leaves were dark-adapted for 30 min before measurement. Minimal fluorescence yield (Fo) was measured with relatively weak measuring light pulses (0.5 μmol m^-2^ s^-1^) at a low frequency (1 Hz). Maximal fluorescence yield (Fm) was determined by applying a pulse saturation light (duration 0.8 s, 1580 μmol m^-2^ s^-1^). Subsequently, the Fv/Fm [Fv/Fm = (Fm—Fo)/Fm] was obtained automatically using ImagingWin software (Version 2.40, Walz). Each treatment includes five replications.

### Chlorophyll Concentration

Total chlorophyll was extracted from frozen leaf samples (~0.2 g) using 80% acetone on a shaker at room temperature until the tissue was completely bleached. The extract was centrifuged at 5,000 *g* for 5 min, and the supernatant was gathered for absorbance measurement at 645 nm and 663 nm using a spectrophotometer (UV-2550, Shimadzu, Japan). Total chlorophyll concentration was calculated using the following formula: total chlorophyll concentration = 20.2A_645_ + 8.02A_663_, as reported by Arnon [21]. The chlorophyll concentration (mg g^-1^ DW) was then calculated.

### Senescence Marker Gene Expression

The eighth leaves that were sampled during the drought progression were used to measure senescence marker gene expression. The sorghum genes that are homologous to known senescence marker genes in *Arabidopsis thaliana* were identified based on data from the National Center for Biotechnology Information (NCBI) database. RNA was isolated and gene expression was analyzed using quantitative RT-PCR, as described by Liu et al. [19], with *Actin1* and *UBC9* as constitutive controls. The genes and the sequences of their specific primers are listed in Table 1.

**Table 1:** Genes and oligonucleotides used in real-time quantitative PCR.

Gene ID	Gene	Primer
Sb01g010030	*Actin1*	F 5′-TGTTCCCTGGGATTGCTG-3′
		R 5′-GCCGGACTCATCGTACTCA-3′
Sb09g023560	*UBC9*	F 5'-TCCATCCGAATATCAACAGCA-3'
		R 5'-GGTCCGTAAGAAGGGAACAAAT-3'
Sb02g022290	*WRKY53*	F 5'-CCTTGCTCCAGTCCTATCTCG-3'
		R 5'-TGCTGCCTCCTCCATTGTTA-3
Sb05g020160	*CHS*	F 5'-TCGGCATCACAGACCATCC-3'
		R 5'-TGAACGCCTCCTCCAACG-3'
Sb01g042450	*GLN1;4*	F 5'-ATGATCGCCGAGACCACC-3'
		R 5'-GGGAACCGAAATCGCAAA-3'
Sb03g032310	*NTR2;5*	F 5'-GCATCGTGCCGTTCGTCT-3'
		R 5'-TCCCCGTCTCCGTCTTGTA-3'
Sb03g027040	*LHCB1;4*	F 5'-TGCAGGCTATCGTCACCG-3'
		R 5'-CCCAAGCTCCCTTCACAAA-3'
Sb05g003480	*RBCS1A*	F 5'-CAACATCAAGCAGACGCAGTG-3'
		R 5'-GCAAAACCGAACGAACAGG-3'

### Carbohydrate Content

Dried leaf samples were ground to a fine powder for carbohydrate analysis. Leaf powder (~0.2 g) was extracted with 6 ml of 80% (v/v) ethanol for 30 min in a water bath at 80°C under agitation and centrifuged at 5,000 *g* for 10 min. The supernatant was then collected. The process was repeated three times. The supernatant was combined and diluted with water to 25 ml; next, 2 ml solution was taken and evaporated in a boiling water bath. The samples were dissolved in 10 ml distilled water, and subsequently the soluble sugars were determined using the anthrone reagent, according to the method of Yemm and Willis [22].

The ethanol-insoluble residue was extracted for starch and measured using the anthrone reagent, according to the method of Clegg [23]. The pellet was re-suspended in 2 ml of water and incubated in a boiling water bath for 15 min. Then, 2 ml 9.2 M perchloric acid was added and stirred for 15 min. The extract solution was centrifuged at 5,000 *g* for 10 min, and the supernatant was collected. The residue was extracted again using perchloric acid and the supernatant was combined and diluted with water to 50 ml. The starch content was then determined using the anthrone reagent.

Total non-structural carbohydrates (TNC) was calculated as the sum of soluble sugars and starch.

### Nitrogen Content

A finely ground leaf sample (~0.1 g) was digested in sulfuric acid with 0.23 g K_2_SO_4_ and 0.07 g CuSO_4_. Nitrogen content was determined by the standard macro-Kjeldahl procedure using a Kjeltec 2300 analyzer unit (Foss Tecator AB, Hoganas, Sweden).

### Soluble Protein and Free Amino Acid Content

Frozen leaf samples (~0.2 g) were homogenized with 10 ml of 50 mM sodium phosphate (pH 7.8) containing 2 mM EDTA and 80 mM L-ascorbic acid. The homogenate was then centrifuged at 15,000 *g* for 20 min and the supernatants were used to determine the soluble protein and free amino acid content. The soluble protein content was measured using the Bradford G-250 reagent [24] and the free amino acid content was determined using the ninhydrin method [25].

### The Effect of Variable Exogenous Sugar and Nitrogen Levels on Detached Leaf Senescence

Sterilized seeds were germinated for 4 d in an incubator at 25°C. After germination, healthy seedlings were transplanted into a plastic container with 5 liters of half-strength Hoagland culture solution, which was renewed every three days, and placed in the growth chamber. The culture solution was continuously aerated, and the pH was adjusted to 6.0 with 0.1 M HCl or 1 M KOH each day. Two weeks after transplanting, the apical 3 cm segments were excised from the third leaves and placed in a Petri dish with 30 ml of test solutions containing 0 or 100 mM glucose and 0.15 or 15 mM N (KNO_3_:NH_4_Cl = 14:1). After incubation at 23°C under dark conditions for 10 hours, 2.19 g mannitol per Petri dish (400 mM, -1.0 MPa) was added to induce osmotic stress, and the samples were incubated for additional time under a 14/10 h day/night cycle at a day/night temperature of 23/15°C. Photosynthetic photon flux density was 600 μmol m^−2^ s^−1^. The amount of chlorophyll degradation was assessed visually each day and chlorophyll was extracted after three days of incubation.

### Statistical Analysis

Data were analyzed by analysis of variance (ANOVA) using SPSS statistics software (Version 19.0 for Windows, SPSS, Chicago, USA). Differences between the mean values were compared using the least significant differences (LSD) post hoc test, and a *P* value ≤ 0.05 was considered significant.

## Results

### Drought Induces Leaf Senescence

**Fig 2:**
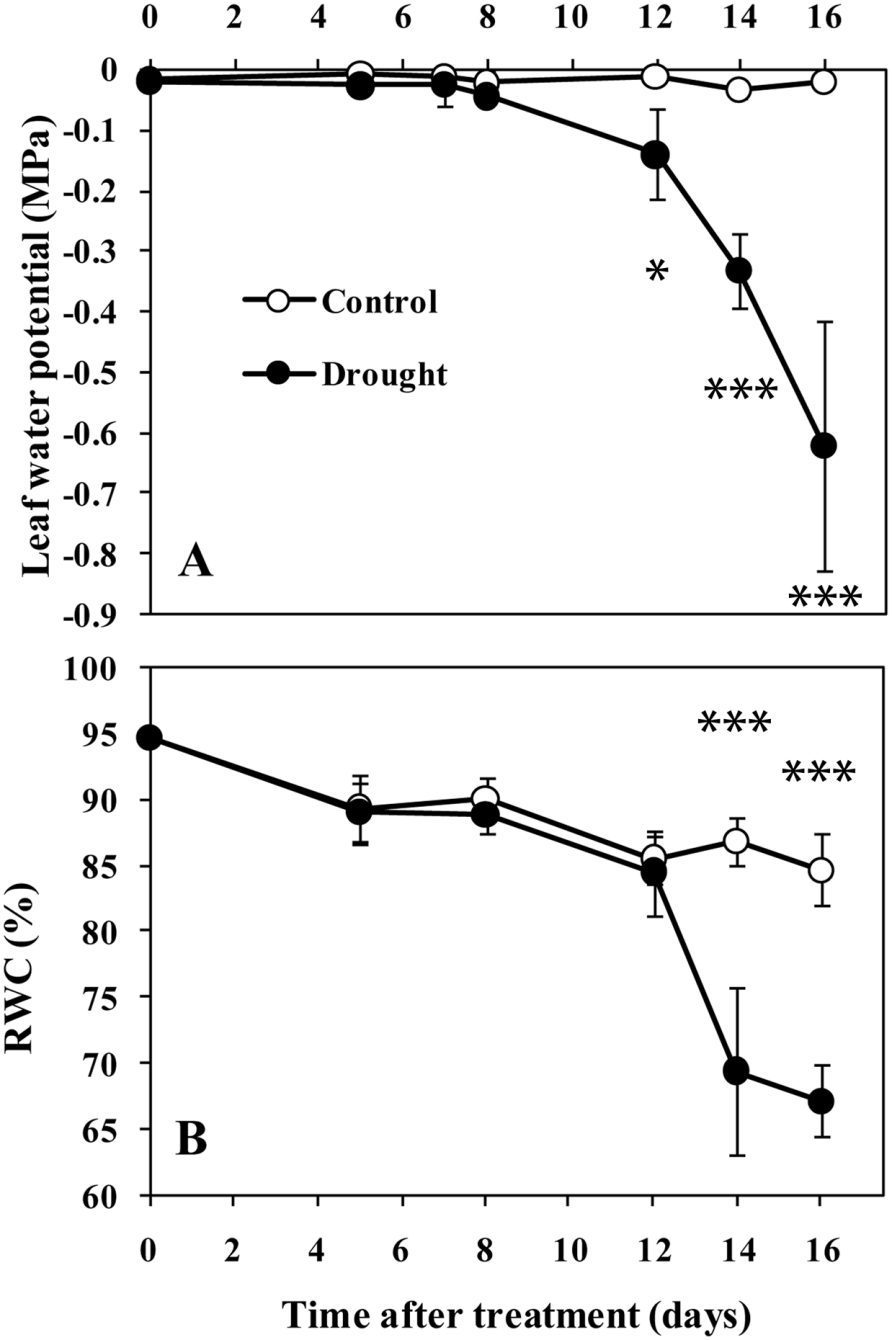
Changes in leaf water potential (A) and relative water content (RWC, B) in sorghum leaf 8 during drought progression. Data represent the mean ± SD (n = 5). Asterisks indicate statistically significant differences between treatments (* *P*≤0.05; ** *P*≤0.01; *** *P*≤0.001).

**Fig 3:**
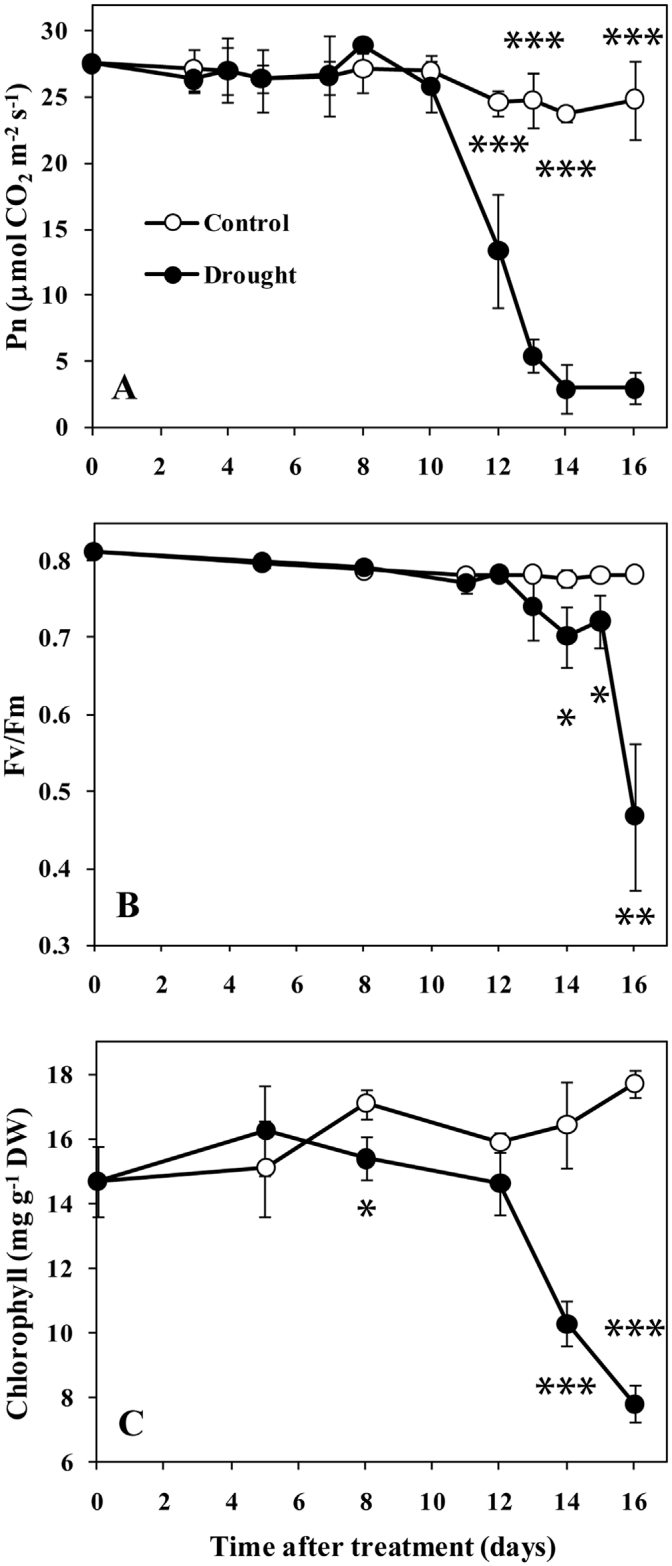
Senescence-dependent changes in sorghum leaf 8 during drought progression. A, Photosynthesis. B, Maximum efficiency of PSII photochemistry (Fv/Fm). C, Chlorophyll content. Data represent the mean ± SD (n = 5). Asterisks indicate statistically significant differences between treatments (* *P*≤0.05; ** *P*≤0.01; *** *P*≤0.001).

Leaf water potential and relative water content was measured to determine leaf water status. Under drought conditions, the water potential was maintained until day 12 and dropped progressively thereafter (Fig 2A). No significant difference in leaf RWC between treatments was seen until day 14, when a substantial drop occurred in the plants under drought conditions (Fig 2B). Several senescence symptoms in the selected eighth leaves were investigated during drought progression to monitor the progression of drought-induced leaf senescence. Under drought conditions, the leaf photosynthesis rate (Pn) decreased sharply from day 12 and remained at a low level thereafter (Fig 3A). No visible change in the Fv/Fm in leaves under drought conditions was seen until day 14. By day 16, the Fv/Fm had fallen to 0.47 (Fig 3B). Similarly, there was no significant difference in leaf chlorophyll content between treatments until day 14, and a substantial decline occurred between days 14 and 16 in the plants under drought conditions (Fig 3C). These results suggest that drought could induce leaf senescence. The symptoms of drought-induced leaf senescence appeared approximately between days 12 and 14 in this study.

**Fig 4:**
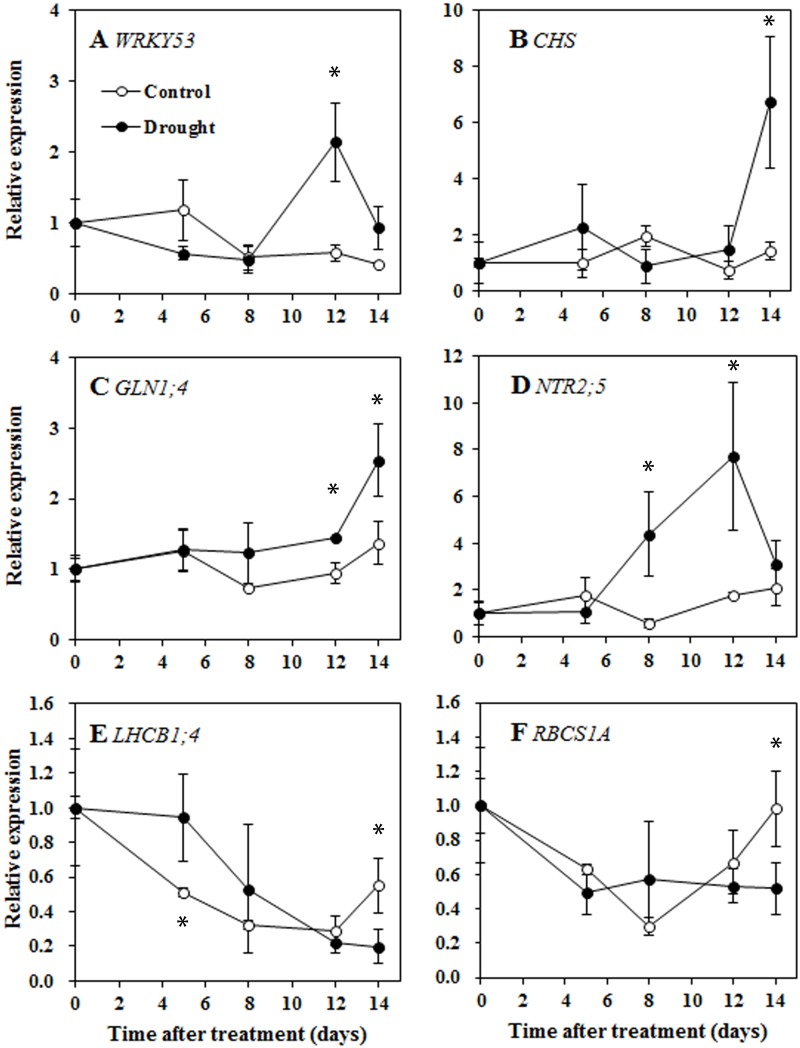
Senescence-dependent changes in gene expression determined by quantitative RT-PCR in sorghum leaf 8 during drought progression. A, *WRKY53*. *B*, *Chalcone synthase* (*CHS*). C, *Cytosolic glutamine synthase1*.*4* (*GLN1;4*). D, *High-affinity nitrate transporter 2*.*5* (*NRT2;5*). E, *Chlorophyll a/b-binding protein* (*LHCB1;4*). F, *Rubisco small subunit 1A* (*RBCS1A*). Expression ratios are presented relative to the values on day 0. Data represent the mean ± SE (n = 3). Asterisks indicate statistically significant differences between treatments (* *P*≤0.05; ** *P*≤0.01; *** *P*≤0.001).

The sorghum genes homologous to known *Arabidopsis thaliana* senescence marker genes were analyzed. As shown in Fig 4, expression of *WRKY53*, an essential senescence-related transcription factor gene [26,27] increased approximately two-fold on day 12. Transcripts of the anthocyanin biosynthetic enzyme *chalcone synthase* (*CHS*) increased at day 14. *Cytosolic glutamine synthase1*.*4* (*GLN1;4*) and *high-affinity nitrate transporter 2*.*5* (*NRT2;5*), both of which are also transcriptional markers induced by N deficiency [28,29], were also transcriptionally activated as the drought conditions progressed. *GLN1;4* transcripts were higher in drought-treated plants than in control plants beginning on day 8 and increased significantly on day 14. *NRT2;5* expression increased from day 8 and peaked at day 12. In addition, the expression of senescence-repressed genes *Chlorophyll a/b-binding protein* (*LHCB1;4*) and *rubisco small subunit 1A* (*RBCS1A*) decreased under both control and drought conditions, but the expression of these genes was lower in drought-treated plants than in control plants by day 14. Overall, the changes in gene expression support the view that drought could induce leaf senescence. The changes in gene expression associated with drought-induced leaf senescence appeared approximately on days 12 to 14 in this study.

### Carbon-Nitrogen Imbalance Is Associated with Drought-Induced Leaf Senescence

**Fig 5:**
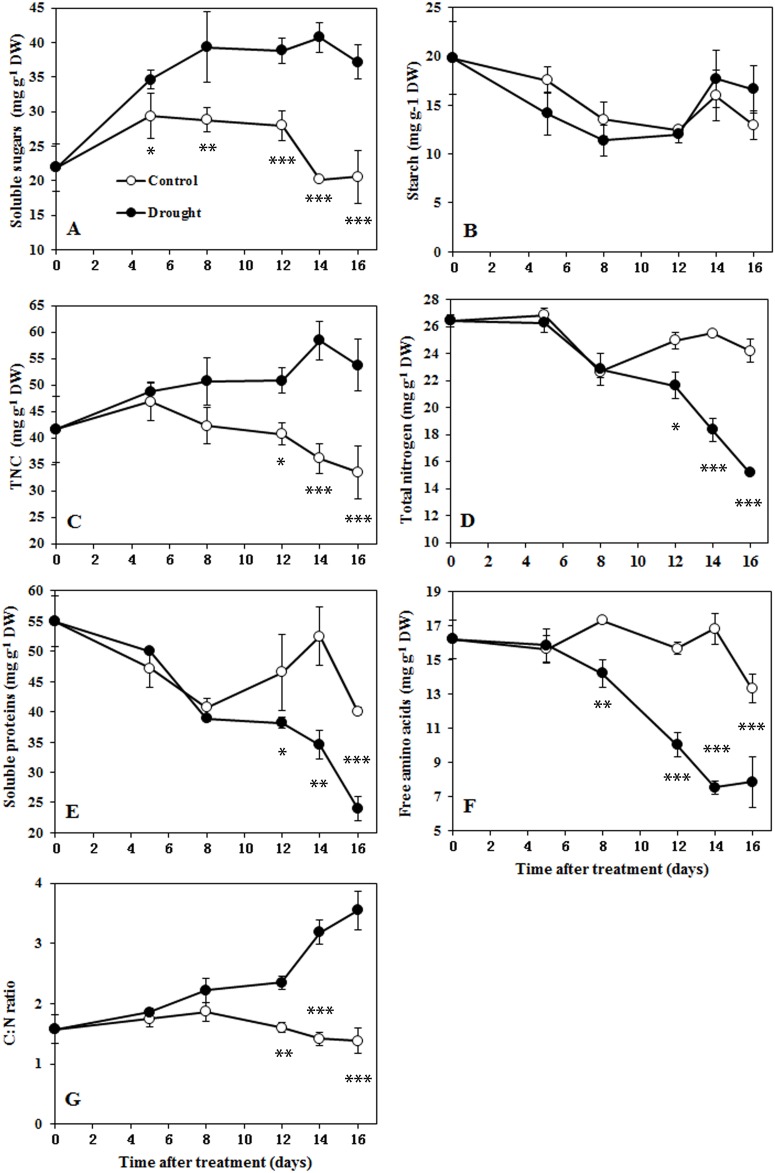
Changes in carbohydrates, nitrogen compounds and the C:N ratio in sorghum leaf 8 during drought progression. A, Soluble sugars. B, Starch. C, Total non-structural sugars. D, Total nitrogen content. E, Soluble proteins. F, Free amino acids. G, C:N ratio. Data represent mean ± SE (n = 3). Asterisks indicate statistically significant differences between treatments (* *P*≤0.05; ** *P*≤0.01; *** *P*≤0.001).

Drought stress positively affected the concentrations of soluble sugars, which increased rapidly from day 5 and remained elevated after day 8 compared with their levels in control plants (Fig 5A). Starch content was less affected by drought: no significant difference in the starch content between treatments was shown during drought progression (Fig 5B). Predictably, the TNC content, which is the sum of soluble sugars and starch, increased to a higher level beginning at day 8, in a manner similar to that of soluble sugars (Fig 5C). In contrast, soil drought negatively affected the concentrations of N compounds, total N content, soluble proteins and free amino acids in drought-treated plants: all of these measurements decreased gradually as the drought progressed (Fig 5D, 5E and 5F). A significant difference in free amino acids between treatments appeared by day 8, and significant differences in total N content and soluble proteins appeared by day 12. The C:N ratio, calculated as the ratio of TNC to total N, was chosen as an indicator of the internal C/N balance as described by Engles [30]. As shown in Fig 5G, the C:N ratio remained relatively constant over time in the control plants. In drought-treated plants, however, the C:N ratio was significantly elevated and increased progressively for the duration of the drought. As expected, a significant difference in C:N ratio between the treatments appeared by day 12 and remained in place from that time on. These results showed that the C/N imbalance that resulted from sugar accumulation and nitrogen deficiency was associated with the drought-induced leaf senescence.

**Fig 6:**
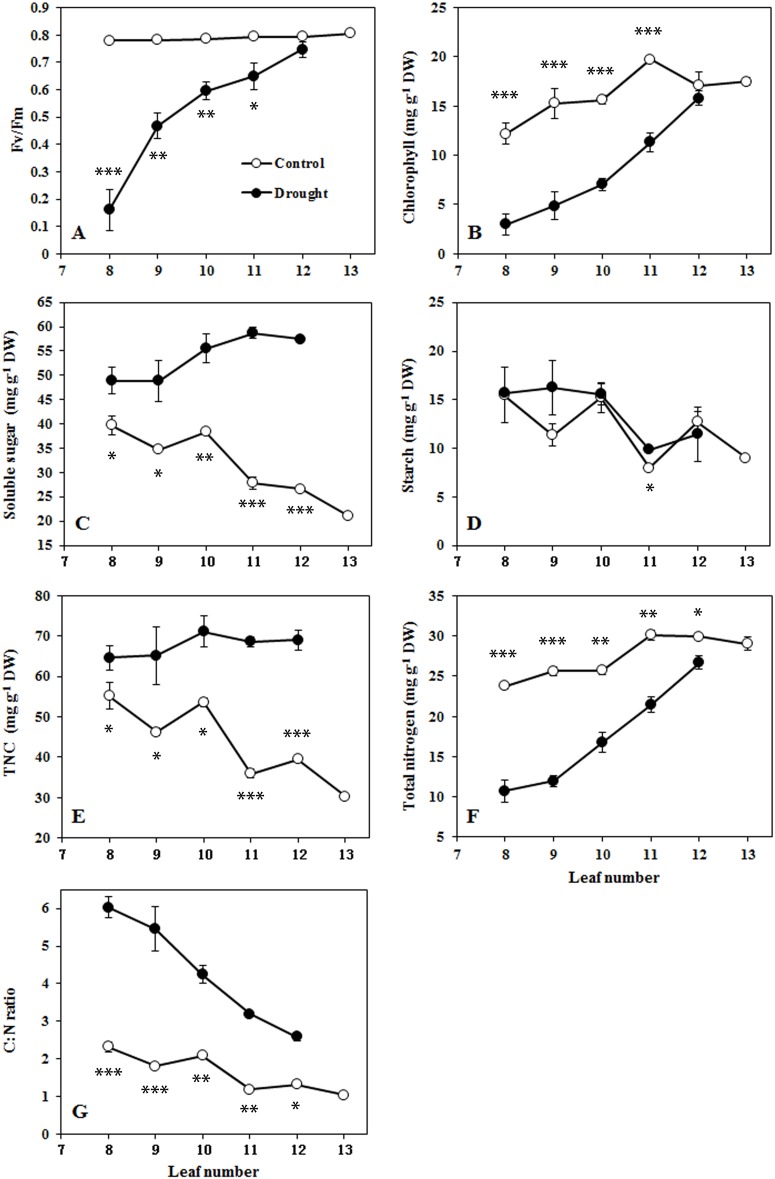
Senescence symptoms and C/N balance in leaves at different positions in the sorghum plant after drought. A, Maximum efficiency of PSII photochemistry (Fv/Fm). B, Chlorophyll content. C, Soluble sugars. D, Starch. E, Total non-structural sugars. F, Total nitrogen content. G, C:N ratio. Data represent the mean ± SE (n = 3). Asterisks indicate statistically significant differences between treatments (* *P*≤0.05; ** *P*≤0.01; *** *P*≤0.001).

The C:N ratio and leaf senescence at different positions in the sorghum plant at the end of the drought treatment were studied. Senescence induced by drought occurred gradually from the bottom to the top of the plant. As shown in Fig 6A and 6B, under drought conditions, both Fv/Fm and chlorophyll content decreased gradually from the youngest, fully-expanded twelfth leaves to the eighth leaves. In well-watered plants, the bottom leaves accumulated more soluble sugars than the top leaves did; in drought-treated plants, however, soluble sugar accumulation was affected differently at different positions so that the bottom leaves accumulated fewer sugars, though sugar levels were still higher in drought-treated plants than in control plants (Fig 6C). Similarly, the starch content was higher in the lower leaves than in the top leaves under control conditions, but the drought had little impact on starch content (Fig 6D). Thus, the bottom leaves contained more TNC in well-watered plants, while all leaves accumulated large quantities of TNC under drought conditions (Fig 6E). The total N contents of leaves at different positions in the sorghum plant are shown in Fig 6F. The bottom leaves accumulated less N compared with the top leaves under control conditions. Soil drought negatively affected the N content, which decreased gradually as leaf age increased from the youngest fully expanded twelfth leaves to the eighth leaves. As shown in Fig 6G, the C:N ratio remained relatively constant with a slight increase in the bottom leaves under control conditions. Drought conditions, on the other hand, stimulated the C:N ratio, which increased progressively as leaf senescence increased from the top leaves to the bottom leaves. These results clearly show that C/N imbalance is associated with drought-induced leaf senescence in a spatial relationship.

### High Sugar and Low Nitrogen Accelerates Senescence Induced by Osmotic Stress in the Detached Leaf

**Fig 7:**
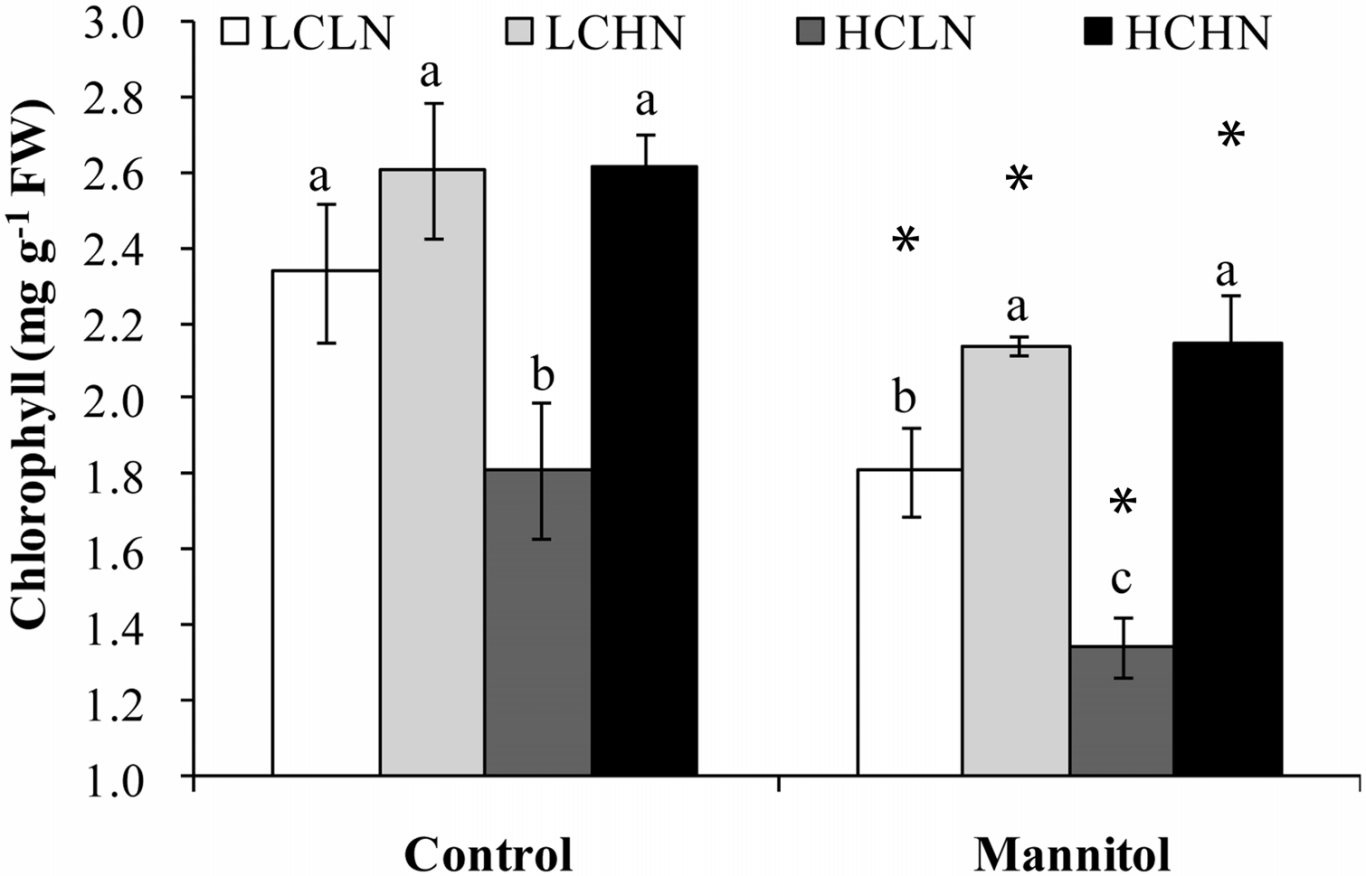
Effects of the C/N level and osmotic stress on chlorophyll content. The chlorophyll content was determined after incubation in test solutions containing 0 or 100 mM glucose and 0.15 or 15 mM N (KNO_3_:NH_4_Cl = 14:1) with or without mannitol (400 mM, -1.0 MPa) for three days. Data represent the mean ± SE (n = 5). Asterisks indicate statistically significant differences between the osmotic stress treatments (*P*≤0.05); different letters indicate differences between the C/N level treatments (*P*≤0.05).

A detached leaf assay was conducted to investigate the effect of changes in the C:N ratio on senescence induced by osmotic stress, a principal component of drought stress. After incubation for three days, the leaf apical segments subjected to high C and low N treatment showed visible chlorosis, and the chlorophyll content declined significantly compared with that in control samples (Fig 7). Osmotic stress caused by mannitol significantly induced leaf senescence, and the chlorophyll content decreased in all four treatments. It is noteworthy that, under osmotic stress conditions, N supplementation alleviated the decrease in chlorophyll content regardless of whether sugar supplementation was also administered, and the leaf apical segments subjected to high C and low N treatment showed a greater decrease in chlorophyll than those subjected to any other treatment. These results suggest that high C and low N status accelerates senescence induced by osmotic stress.

## Discussion

Together, chlorophyll loss and photochemical efficiency constitute a convenient assay for chloroplast senescence [26]. Leaf senescence is accompanied by decreased expression of genes related to photosynthesis and increased expression of senescence-associated genes (SAGs) [26]. In this study, leaf senescence occurred approximately 12 days after drought treatment began, and various senescence symptoms, including decreasing photosynthesis, PSII photochemistry efficiency, chlorophyll content and the differential expression of senescence marker genes, were observed. The C:N ratio was significantly increased between days 10 and 12 after drought treatment (Fig 5). These results showed that leaf senescence occurred in association with an increased C:N ratio. In addition, leaf senescence occurred in the older (lower) leaves, which had higher C:N ratios, and did not occur in the younger (upper) leaves, which had lower C:N ratios (Fig 6). Therefore, the occurrence of leaf senescence is strongly related to the C/N imbalance under drought stress. Similarly, C/N imbalance also preceded natural leaf senescence in mastic tree (*P*. *lentiscus*) under Mediterranean field conditions [31].

Leaf senescence is a tightly regulated physiological process that is basically governed by developmental age. Leaf senescence can be induced or accelerated by environmental stresses such as drought, heat and salinity, as it contributes to plant survival under stress conditions [2]. Under drought stress, leaves show symptoms of senescence in association with sugar accumulation and N deprivation [11,13]. Sugars act as signaling molecules during various stages of plant development and for diverse physiological functions [32]. When sugar levels exceed acceptable thresholds, they inactivate photosynthetic activity and trigger leaf senescence [33]. The sugar metabolic rate affects leaf longevity and regulates the developmental senescence process [2]. Drought generally results in the accumulation of soluble sugar, which is considered to be one of the most important osmolytes that contributes to osmotic adjustments. Therefore, sugar signaling is likely to play a role in the senescence response to drought stress [32]. However, drought causes sugars to accumulate in leaves of all ages and at all positions, while drought-induced senescence occurred in the bottom leaves only (Fig 6). Therefore, carbohydrate accumulation is necessary but not sufficient to induce leaf senescence under drought conditions.

Leaf senescence can be induced by a low nutrient supply, especially a low N supply [34, 35]. In barley and *Arabidopsis*, N deprivation resulted in accelerated leaf senescence. When additional NO_3_^-^ was supplied at the start of senescence, senescence could be halted or even reversed [36]. As in previous studies, drought gradually decreased the N levels, and bottom leaves had lower levels than upper leaves after drought stress. N levels tended to have the most powerful effect on leaf senescence. However, in detached leaves, a low N supply with high sugar levels was associated with more severe leaf senescence than a low N supply with lower sugar levels during drought stress (Fig 7). Interestingly, N deficiency often results in sugar accumulation in the leaf [35]. In addition, an exogenous glucose supply leads to more accumulation of sugars during senescence in *Arabidopsis* plants grown with low N levels than in plants grown with high N levels [37]. Therefore, it is likely that the role of N deprivation in inducing leaf senescence is at least partly a result of sugar accumulation, suggesting that N deprivation alone is not the critical element in inducing leaf senescence under drought stress.

In addition, C/N balance, the ratio of C to N metabolites in the cell, is one of the most important factors in the regulation of plant metabolism, growth and development, though C and N are each important in their own right as well [6,7,8]. It has been reported that the C/N balance rather than C or N alone played an important role in regulating leaf senescence under normal conditions, and that leaf senescence can be triggered by a higher C:N ratio [7,10]. It is well known that drought generally results in sugar accumulation and N deficiency, leading to high sugar and low N levels in mature leaves with leaf senescence. In this study, exogenous application of excess sugar combined with limited N also promoted chlorophyll loss in osmotically stressed leaf segments (Fig 7). This result suggested that high C and low N status accelerates senescence induced by osmotic stress, a principal component of drought stress. It is worth noting that osmotic stress does not reflect natural drought stress entirely, but it is hard to tightly control C and N status in the undetached leaves of plants under natural drought stress. Therefore, the finding that C/N imbalance promotes drought-induced leaf senescence under drought stress needs to be confirmed further.

Most previous work on the regulation of drought-induced leaf senescence has focused on the roles of phytohormones, especially CK and ABA [3,4], as well as reactive oxygen species (ROS) [5]. In the present study, we showed that C/N balance may also be involved in the regulation of drought-induced leaf senescence in *Sorghum bicolor*. CKs are the strongest senescence-delaying hormones [2]. A CK-mediated delay in senescence is correlated with extracellular invertase activity [38]. When extracellular invertase activity is inhibited, the CK-mediated delay in leaf senescence is also inhibited. The results showed that extracellular invertase plays a role in mediating CK’s action in delaying leaf senescence, suggesting that carbohydrate partitioning associated with invertase activity may be related to a CK-mediated delay of leaf senescence. High levels of CKs delayed drought-induced leaf senescence and enhanced drought tolerance [3]. Stress-induced CK synthesis also promoted drought tolerance through a CK-dependent coordinated regulation of C and N metabolism, which contributes to the cellular C/N balance [12]. Overall, these results suggest a link among CK action, C/N balance and drought-induced senescence.

ABA is a key plant hormone that mediates plant responses to environmental stresses [39]. It is also considered to be an enhancer of leaf senescence [2]. Drought-induced ABA was positively and significantly correlated with C remobilization from senescing leaves to grains in wheat plants subjected to drought stress [4]. Wingler [31] proposed that the senescence response to abiotic stress is likely to be dependent on sugar and ABA signaling and the interactions between them. ABA synthesis and signaling have also been identified as important components in sugar signaling [40]. These results suggest a link among ABA, C/N balance and drought-induced senescence. Taken together, C/N balance may regulate drought-induced leaf senescence through interaction with CK and ABA.

Furthermore, it is thought that several abiotic stresses, including drought, induce a reduction in CO_2_ assimilation rates with a consequent increase in reactive oxygen species (ROS) production in the chloroplasts, ultimately leading to leaf senescence [41]. High concentrations of sugars inactivate photosynthetic activity [2]. In this study, inhibition of photosynthesis was accompanied by an increase in the C:N ratio. This suggested that C/N imbalance may induce the photo-oxidation mediated by the inhibition of photosynthesis, ultimately leading to leaf senescence. In addition, it is interesting to note that changes in the C:N ratio occurred well before chlorophyll loss, and that both changes in the C:N ratio and chlorophyll loss occurred well before the decrease in Fv/Fm in the present study. It seems that chlorophyll is more sensitive to C/N imbalance than Fv/Fm is. One possible reason for this is that there was a chlorophyll loss for N remobilization caused by the C/N imbalance before the Fv/Fm and a second chlorophyll loss caused by the photo-oxidation. Correspondingly, a similar trend in which chlorophyll loss occurred well before the decrease in Fv/Fm was also observed during natural leaf senescence in *Arabidopsis* [42] and mastic tree [31], chilling-induced leaf senescence in *Arabis alpine* [43] and drought-induced leaf senescence in *Salvia officinalis* [44]. This suggests that C/N balance may play a ubiquitous role in leaf senescence.

In conclusion, the present study suggests that C/N balance may be also involved in the regulation of drought-induced leaf senescence. It provides further evidence to support the regulation of leaf senescence by the C/N balance in plants and extends our current understanding of drought-induced leaf senescence regulation. To understand the molecular mechanism underlying the regulation of drought-induced senescence, the signaling components involved in the C/N balance and the interaction of C/N balance with phytohormones and ROS in plants should be studied in detail.

## Supporting Information

S1 Dataset S1 Dataset contains data on soil water content, leaf water potential, relative leaf water content, photosynthesis, maximum efficiency of PSII photochemistry (Fv/Fm), chlorophyll content, gene expression, carbohydrates, nitrogen compounds and the C:N ratio in the eighth sorghum leaf during drought progression. (XLSX)

S2 Dataset S2 Dataset contains data on maximum efficiency of PSII photochemistry (Fv/Fm), chlorophyll content, carbohydrates, nitrogen and the C:N ratio in leaves at different positions in the sorghum plant after drought. (XLSX)

S3 Dataset S3 Dataset contains data on chlorophyll content under variable exogenous C/N levels. (XLSX)
